# Supplementary material for: Prevalence, predictors, and economic burden of mental health disorders among asylum seekers, refugees and migrants from African countries: A scoping review
Source: PLoS One. 2024 Jun 24;19(6):e0305495. doi: 10.1371/journal.pone.0305495 (PMC11195976; doi:10.1371/journal.pone.0305495)
Supplement: S1 File — (DOCX) [file pone.0305495.s001.docx]

**Supporting Information**

**Manuscript: Prevalence, Predictors, and Economic Burden of Mental Health Disorders Among Asylum Seekers, Refugees and Migrants from African Countries**

Wael Osman et al.

**S1: Definitions/Terms:**

Glossary of migration from International Migration Law No. 34 - Glossary on Migration

| Term | Meaning |
| --- | --- |
| Asylum seeker | An individual who is seeking international protection. In countries with individualized procedures, an asylum seeker is someone whose claim has not yet been finally decided on by the country in which he or she has submitted it. Not every asylum seeker will ultimately be recognized as a refugee, but every recognized refugee is initially an asylum seeker. |
| Migrant | At the international level, no universally accepted definition for “migrant” exists. We choose to concur with the definition of the International Organization of Migration: “any person who is moving or has moved across an international border or within a State away from his/her habitual place of residence, regardless of (1) the person’s legal status; (2) whether the movement is voluntary or involuntary; (3) what the causes for the movement are; or (4) what the length of the stay is.” |
| Refugee | A person who, “owing to a well-founded fear of persecution for reasons of race, religion, nationality, membership of a particular social group or political opinions, is outside the country of his nationality and is unable or, owing to such fear, is unwilling to avail himself of the protection of that country.” |
| Undocumented migrant | A non-national who enters or stays in a country without the appropriate documentation. This includes, among others: a person (a) who has no legal documentation to enter a country but manages to enter clandestinely, (b) who enters or stays using fraudulent documentation, (c) who, after entering using legal documentation, has stayed beyond the time authorized or otherwise violated the terms of entry and remained without authorization |
| Forced migration | A migratory movement which, although the drivers can be diverse, involves force, compulsion, or coercion (IOM). |
| Prevalence | Is the proportion of a population who have a specific characteristic in a given time period (NIH, USA)  The number of cases of a disease, number of infected people, or number of people with some other attribute present during a particular interval of time (CDC, USA) |
| Predictors | In epidemiology, "predictor" and "risk factor" are interchangeable. A "predictor" is rather relevant in prediction modeling, whereas "risk factor" is relevant in etiology questions. The risk factor itself is an event, circumstance, or characteristic that is common in people suffering from a particular disease. Predictors are circumstances, characteristics, or events that occur during an action, that may favor one or more outcomes. The use of "risk factors" preconceived especially in case control or cohort studies and what we do is reaccreting the association using RR; when several factors are assessed, confounding is adjusted, and population-specific predictors are identified, the predictor terminology becomes more relevant.  R Kaaja (2008), Mario Tumbarello et al. (2002). |
| Economic burden | In the context of mental health, economic burden encompasses both direct costs of treated and untreated mental illness, as well as indirect costs such as reduced labor supply, incarceration costs, and homeless shelter costs.  Heather L. Taylor, JAMA, 2023. |
