# Supplementary material for: Prevalence, predictors, and economic burden of mental health disorders among asylum seekers, refugees and migrants from African countries: A scoping review
Source: PLoS One. 2024 Jun 24;19(6):e0305495. doi: 10.1371/journal.pone.0305495 (PMC11195976; doi:10.1371/journal.pone.0305495)
Supplement: S2 File — (DOCX) [file pone.0305495.s002.docx]

**Supporting Information**

**Manuscript: Prevalence, Predictors, and Economic Burden of Mental Health Disorders Among Asylum Seekers, Refugees and Migrants from African Countries**

Wael Osman et al.

**S2: The search strategy:**

- Defining “Asylum seekers, refugees and migrants” in accordance with the international migration law in the table below.
- Defining mental health disorders in accordance with the DSM V manual. Common MHD such as depression, anxiety, post-traumatic stress disorder, schizophrenia, bipolar disorder, psychosis etc. will be added by name to the search strategy.
- 2 search strings (one focusing on prevalence and predictors; the other focusing on economic burden)

**1^st^ search string**: Prevalence and predictors of mental health disorders among asylum seekers, refugees, and migrants from African countries

| Prevalence / predictors | “prevalen*” OR “predictor*” OR “cause*” OR “risk factor” |
| --- | --- |
|  | AND |
| Mental health disorders | "Mental Disorders" OR "Suicide" OR “mental dis*” OR “mental ill*” OR “mentally ill*” OR “mental health” OR “mood disorder*” OR “depress*” OR “anxiety” OR “bipolar” OR “PTSD” OR “psychosis” OR “psychotic” OR “schizophren*” OR “post-traumatic stress” OR “posttraumatic stress” OR “suicid*” |
|  | AND |
| Asylum seekers / Refugees / Migrants | ("migrant*" OR "migrant workers" OR "undocumented migrant*" OR "illegal migrant*" OR "economic migrant*" OR "refugee*" OR "asylum seeker*" OR "asylees" OR "forced migrant*" OR "stateless person*" OR "refugee camp resident*" OR "immigrant*" OR "exile*") |
|  | AND |
| Africa | "Afric*" OR "Algeria" OR "Angola" OR "Benin" OR "Botswana" OR "Burkina Faso" OR "Burundi" OR "Cabo Verde" OR "Cameroon" OR "Central African Republic" OR "Chad" OR "Comoros" OR "Congo" OR "Cote d'Ivoire" OR "Democratic Republic of the Congo" OR "Djibouti" OR "Egypt" OR "Equatorial Guinea" OR "Eritrea" OR "Eswatini" OR "Ethiopia" OR "Gabon" OR "Gambia" OR "Ghana" OR "Guinea" OR "Guinea-Bissau" OR "Kenya" OR "Lesotho" OR "Liberia" OR "Libya" OR "Madagascar" OR "Malawi" OR "Mali" OR "Mauritania" OR "Mauritius" OR "Morocco" OR "Mozambique" OR "Namibia" OR "Niger" OR "Nigeria" OR "Rwanda" OR "Sao Tome and Principe" OR "Senegal" OR "Seychelles" OR "Sierra Leone" OR "Somalia" OR "South Africa" OR "South Sudan" OR "Sudan" OR "Tanzania" OR "Togo" OR "Tunisia" OR "Uganda" OR "Zambia" OR "Zimbabwe" |

**2^nd^ search string:** Economic burden of mental health disorders among asylum seekers, refugees and migrants from African countries

| Economic Burden | "economic burden" OR "financial burden" OR "economic cost" OR "financial cost" OR "economic impact" OR "financial impact" OR "economic strain" OR "financial strain" OR "economic stress" OR "financial stress" OR "economic expense" OR "financial expense" |
| --- | --- |
|  | AND |
| Mental health disorders | "Mental Disorders" OR "Suicide" OR “mental dis*” OR “mental ill*” OR “mentally ill*” OR “mental health” OR “mood disorder*” OR “depress*” OR “anxiety” OR “bipolar” OR “PTSD” OR “psychosis” OR “psychotic” OR “schizophren*” OR “post-traumatic stress” OR “posttraumatic stress” OR “suicid*” |
|  | AND |
| Asylum seekers / Refugees / Migrants | ("migrant*" OR "migrant workers" OR "undocumented migrant*" OR "illegal migrant*" OR "economic migrant*" OR "refugee*" OR "asylum seeker*" OR "asylees" OR "forced migrant*" OR "stateless person*" OR "refugee camp resident*" OR "immigrant*" OR "exile*") |
|  | AND |
| Africa | "Afric*"OR "Algeria" OR "Angola" OR "Benin" OR "Botswana" OR "Burkina Faso" OR "Burundi" OR "Cabo Verde" OR "Cameroon" OR "Central African Republic" OR "Chad" OR "Comoros" OR "Congo" OR "Cote d'Ivoire" OR "Democratic Republic of the Congo" OR "Djibouti" OR "Egypt" OR "Equatorial Guinea" OR "Eritrea" OR "Eswatini" OR "Ethiopia" OR "Gabon" OR "Gambia" OR "Ghana" OR "Guinea" OR "Guinea-Bissau" OR "Kenya" OR "Lesotho" OR "Liberia" OR "Libya" OR "Madagascar" OR "Malawi" OR "Mali" OR "Mauritania" OR "Mauritius" OR "Morocco" OR "Mozambique" OR "Namibia" OR "Niger" OR "Nigeria" OR "Rwanda" OR "Sao Tome and Principe" OR "Senegal" OR "Seychelles" OR "Sierra Leone" OR "Somalia" OR "South Africa" OR "South Sudan" OR "Sudan" OR "Tanzania" OR "Togo" OR "Tunisia" OR "Uganda" OR "Zambia" OR "Zimbabwe") |
